# Heritability and Correlation Estimates for Serum Insulin-like Growth Factor I Concentration, Weight, Weight Gain, and Height in Angus Beef Cattle in a Long-Term Divergent Selection Study for Serum Insulin-like Growth Factor I (1989 to 2017)

**DOI:** 10.3390/ani14233548

**Published:** 2024-12-09

**Authors:** Michael E. Davis, Rosalia C. M. Simmen

**Affiliations:** 1 Department of Animal Sciences, The Ohio State University, 2027 Coffey Road, Columbus, OH 43210, USA; 2 Department of Physiology & Cell Biology, University of Arkansas for Medical Sciences, 4301 West Markham Street, Little Rock, AR 72205, USA; simmenrosalia@uams.edu

**Keywords:** beef cattle, IGF-I, genetic parameters, growth traits

## Abstract

This study investigated the genetic parameters for serum IGF-I concentrations and growth traits in beef cattle. A divergent selection experiment for serum IGF-I concentration was initiated in 1989. One hundred spring-calving (50 high line and 50 low line) and 100 fall-calving (50 high line and 50 low line) black Angus cows with unknown IGF-I concentrations were randomly assigned to the two divergent selection lines. For the 2009 through 2017 breeding seasons, the selection criterion in the two lines was changed from serum IGF-I concentration to high vs. low maintenance energy expected progeny differences (ME EPDs). The number of records available for analysis varied from 2056 for IGF-I concentration on day 42 of the postweaning performance test to 2988 for birth weight, with the exception that only 617 records were available for IGF-I concentration at weaning. (Co)variance components were estimated for direct and maternal additive genetic effects using an animal model and multiple-trait, derivative-free, restricted maximum likelihood (MTDFREML) computer programs. Direct heritability estimates for serum IGF-I concentrations ranged from 0.34 ± 0.07 to 0.46 ± 0.07, whereas direct heritability estimates for weight traits ranged from 0.32 ± 0.05 to 0.39 ± 0.07. Maternal genetic effects on IGF-I were low, with estimates ranging from 0.02 ± 0.11 to 0.17 ± 0.05, and were similarly low for weight traits. Maternal permanent environmental effects were negligible for IGF-I but were larger for weight traits, especially for weaning weight (0.22 ± 0.04). Direct maternal correlations for postweaning IGF-I concentrations were approximately −0.90. The genetic correlations of IGF-I with weight traits were generally low, with negative correlations observed for birth weight and small positive correlations for the other weight traits. Environmental and phenotypic correlations between IGF-I concentrations and growth traits were generally small, indicating circulating IGF-I has a modest impact on growth traits in beef cattle.

## Simple Summary

This study aimed to estimate genetic parameters for serum insulin-like growth factor I concentrations and their correlations with performance traits in beef cattle. A divergent selection experiment for this important hormone began in 1989. From the 2009 through to the 2017 breeding season, the selection criterion in the two lines was changed to high vs. low maintenance energy expected progeny differences. Estimates of direct heritability were moderate, ranging from 0.34 ± 0.07 to 0.46 ± 0.07 for insulin-like growth factor I concentrations and from 0.32 ± 0.05 to 0.39 ± 0.07 for the growth traits. The heritability of maternal genetic effects for insulin-like growth factor I concentration ranged from 0.02 ± 0.11 to 0.17 ± 0.05 and was similarly low for the weight traits. Maternal permanent environmental effects were negligible for insulin-like growth factor I but were somewhat larger for the weight traits. Direct maternal correlations for postweaning insulin-like growth factor I concentrations were approximately −0.90. The genetic correlations of insulin-like growth factor I with weight traits were generally positive but small. Environmental and phenotypic correlations between insulin-like growth factor I concentrations and growth traits were generally low, indicating that shared environmental influences are minimal, and that this hormone has a modest impact on growth traits. These findings suggest that while IGF-I concentration has some influence, it is not the primary determinant of weight gain or hip height.

## 1. Introduction

Insulin-like growth factor I (IGF-I) is an important hormone that regulates animal growth and development. As a mediator of growth hormone (GH) effects, IGF-I promotes cell proliferation and differentiation. Research in mice demonstrated the heritability of serum IGF-I concentration and its significant impact on growth performance. A heritability estimate of 0.40 ± 0.27 for plasma IGF-I concentration at 35 days of age was reported in mice [1]. The relationship of circulating IGF-I concentration with body size and growth rate justified its use as a marker for growth in selection experiments. Mouse selection experiments [2,3,4,5] indicated that selection for increased serum IGF-I concentration results in increased weights and gains, and selection for decreased IGF-I results in reduced weights and gains. Furthermore, elimination of circulating IGF-I through gene deletion in mice caused severe growth retardation [6]. IGF-I −/− mice exhibited virtually no GH-induced growth, suggesting that IGF-I mediates the effects of GH on body growth [7]. The Cre/loxP recombination system was used to delete the IGF-I gene in mouse livers, with a 75% reduction in serum IGF-I concentration [6,8]. Despite this reduction, there was little effect on postnatal body growth, leading to the conclusion that IGF-I influences body growth primarily through autocrine/paracrine mechanisms rather than via endocrine mechanisms.

Understanding the role of IGF-I in beef cattle is particularly significant due to its potential to enhance growth rates and overall productivity. Despite its importance, limited research exists on the practical application of IGF-I as a selection criterion in cattle breeding programs. To address this gap, a divergent selection experiment was initiated in 1989 at the Eastern Agricultural Research Station (EARS), Belle Valley, Ohio, USA, focusing on serum IGF-I concentration in Angus cattle. Therefore, the primary objectives of this study were to evaluate the utility of IGF-I as a biological indicator trait for growth in beef cattle and to elucidate the underlying biological mechanisms. We hypothesized that selection for higher serum IGF-I concentration would lead to improved growth performance. This research project aims to contribute to the field of animal breeding and genetics by providing insights that will enhance beef cattle production.

## 2. Materials and Methods

### 2.1. Selection Procedures

Divergent selection for serum IGF-I concentration began in 1989 at the Eastern Agricultural Research Station (EARS) in Belle Valley, OH, USA. Two-hundred purebred black Angus cows were used to establish the high and low selection lines. The cows were divided into 100 spring-calving (50 high line, 50 low line) and 100 fall-calving (50 high line, 50 low line) groups with unknown IGF-I concentrations. Cows from the initial base population were randomly assigned to either the high or low selection line.

The procedures for this selection experiment were described previously [9]. A breeding program was implemented using bulls with the highest and lowest adjusted residuals for serum IGF-I concentrations. Each year, the four bull calves with the highest and lowest residuals for serum IGF-I concentrations were retained for breeding in their respective lines. The residuals were adjusted for the age of the calf (in days) and age of the dam (in years), and selection was based on the average of three serum IGF-I measurements taken at days 28, 42, and 56 of a 140-day postweaning performance test (denoted as IGF28, IGF42, and IGF56). Bulls were selected for breeding only if they were classified as “satisfactory” in a post-test breeding soundness exam. To minimize inbreeding, no more than two sons from the same sire or dam were retained for breeding. Yearling bulls were used each breeding season to reduce the generation interval.

Approximately eight cows were culled annually from each selection line due to physical unsoundness, failure to conceive, or age. They were replaced with about eight pregnant heifers showing the highest or lowest adjusted residuals for serum IGF-I concentration. All available heifers were bred, with selection based on the average IGF-I concentration from three blood samples collected at the same time points as for the bulls (days 28, 42, and 56 of the postweaning test) [9]. Serum IGF-I concentration on the day of weaning was also determined for each calf beginning with the 2003 calf crop, as research at that time by Australian scientists [10] indicated that this trait might be an effective selection criterion.

Selection pressure was relaxed in the 2007 and 2008 breeding seasons. Selection for IGF-I concentration was discontinued, and cows and heifers were mated to either Angus or Simmental bulls. Beginning with the 2009 breeding season, the selection criterion in the IGF-I selection lines was changed from serum IGF-I concentration to maintenance energy expected progeny difference (ME EPD), as provided by the Red Angus Association of America. Cows and heifers in the high IGF-I selection line were mated to bulls with high ME EPDs, and cows and heifers in the low IGF-I line were mated to low ME EPD bulls.

### 2.2. Mating Scheme

High IGF-I line heifers were stratified such that each bull was assigned one heifer from the top quartile of IGF-I concentrations, one from the next quartile, and so on. The same approach was used for the low IGF-I line, with males and females presenting the lowest values. This mating strategy aimed to increase the likelihood of producing at least one replacement bull and two replacement heifers from each sire annually. Cows aged two years and older were randomly paired with bulls for mating. To minimize inbreeding, mating between half-sibs and closer relatives was avoided for both heifers and cows.

In the 2009 breeding season and onward, females in the high line were mated to one of three high (undesirable) ME EPD Red Angus bulls, and cows and heifers in the low line were mated to one of three low (desirable) ME EPD Red Angus bulls using artificial insemination. Semen from these bulls was purchased from artificial insemination cooperatives in the United States. Using a timed insemination protocol, cows and heifers were bred for two estrous cycles. The first calves produced in this portion of the project were born in the spring 2010 calving season. The long-term selection experiment was concluded following the weaning of the 2017 calf crop.

### 2.3. Management Procedures

Dams raised spring-born calves without creep feed until weaning at about seven months of age. After weaning, bull calves were provided ad libitum access to a corn–soybean meal-based concentrate diet, plus 2.3 kg of grass hay per bull daily. Heifer calves born from spring 1989 to fall 1993 were given ad libitum access to nonprotein nitrogen-treated corn silage and grass hay in large round bales for a two-week adjustment period and a 140-day test period. Heifers born in the spring of 1994 and onward were fed a corn–soybean meal diet designed to achieve postweaning gains of approximately 0.75 kg/day. Bulls were housed in a three-sided barn with adjoining exercise lots at EARS. Heifers born from spring 1989 to fall 1993 were housed in a dry lot with access to an enclosed barn at the North Appalachian Experimental Watershed (NAEW) in Coshocton, OH, USA. Heifers born in the spring of 1994 and onward were housed in a three-sided barn with adjoining exercise lots at EARS.

Fall-born calves were weaned at an average age of approximately 140 days and then fed a corn–soybean meal concentrate diet designed to achieve gains of about 0.9 kg/day, along with grass hay, in a dry lot for 112 days. After this growing period, bull calves remained at EARS and were managed similarly to the spring-born bulls. Heifer calves born in the fall of 1993 or earlier were transported to NAEW and managed like spring-born heifers. Heifers born in the fall of 1994 and later stayed at EARS and were fed the same diet as the spring-born heifers.

### 2.4. Data Collection

From 1989 through to the spring of 2003, all bull and heifer calves were weighed at birth, weaning, the beginning of the postweaning performance test, and every 28 days after that until the conclusion of the 140-day postweaning period. In addition, all calves born from 1990 through to the spring of 2003 were weighed on day 42 of the postweaning test, when one of the three blood samples was collected.

Calves born in the fall of 2003 and 2004 (except for a small number of replacement heifers who remained at EARS) were transported to Texas A&M University for assessment of residual feed intake of calves in the high and low IGF-I selection lines. Therefore, only birth weights were available for these calves. The fall-calving cow herd was dispersed following the birth of the calves in 2004. Only the spring-calving cow herd remained at EARS for the duration of the study. Beginning with the calves born in the fall of 2003, blood samples were collected on the day of weaning to determine serum IGF-I concentrations. Calves born from 2005 through to 2017 were sold following weaning. Therefore, the only data available for these calves were birth weight, weaning weight, and IGF-I concentration at weaning. The number of records was 617, 2123, 2056, and 2126 for IGF-I concentration at weaning and at day 28, 42, and 56, respectively, of the postweaning period, and 2180 for the mean IGF-I concentration of each calf. Fewer observations were available on day 42 because weights and blood samples were not taken at that time for calves born in the spring of 1989. In addition, serum samples for heifers born in the spring of 1990 were damaged by a freezer malfunction, which necessitated resampling heifers at days 84, 98, and 112 of the postweaning period. The day 84, 98, and 112 IGF-I values were used to calculate the mean IGF-I concentrations of the spring 1990 heifers. Therefore, 2180 observations were available for mean IGF-I concentration, but fewer observations were available for IGF-I concentration on days 28, 42, and 56. In addition, on rare occasions, glass test tubes were broken during centrifugation, contributing to the differing numbers of observations for IGF-I concentration at days 28, 42, and 56, as well as for the mean IGF-I values. The number of observations for the performance traits ranged from 2057 for weight at day 42 to 2988 for birth weight. The average age of calves included in this study was 216 days at weaning and 252 days at the beginning of the postweaning test.

### 2.5. Serum Samples

At each sampling date, approximately 25 mL of blood was collected from each animal via jugular puncture and was added to sterile 16 mm × 150 mm glass tubes. The blood was allowed to clot for 24 h at 4 °C. Then, serum was obtained by centrifugation at 1800× *g* for 20 min and frozen at −20 °C until assayed for IGF-I.

### 2.6. Insulin-like Growth Factor I Assays

Serum IGF-I concentrations were determined in Dr. R.C.M. Simmen’s laboratory at the University of Florida from 1989 to 2003 using a radioimmunoassay described previously [11]. Briefly, binding proteins were removed by acid-ethanol extraction, and each sample was diluted 1:10 in assay buffer and assayed in duplicate using human recombinant IGF-I as the standard and iodinated tracer. Antisera raised in rabbits against human IGF-I (UBK487) were used at a 1:18,000 dilution. Antigen-antibody complexes were precipitated with goat anti-rabbit gamma globulin and normal rabbit serum. IGF-I concentrations were calculated from the standard curve.

From 2004 through to 2017, blood was collected from each calf and placed on absorbent paper cards. The cards were allowed to dry before being sent to Primegro (Rivalea, Corowa, New South Wales, Australia). An Enzyme-linked Immunosorbent Assay (ELISA) patented and licensed to Primegro was used to determine the concentration of IGF-I in the blood.

### 2.7. Statistical Analysis

Data were analyzed using multiple-trait, derivative-free, restricted maximum likelihood (MTDFREML) computer programs [12]. The pedigree file of 3767 animals consisted of the animals in the herd at the start of the experiment, their three-generation pedigrees, and all animals subsequently produced in the selection experiment. It was used to derive the relationship matrix. Each trait was analyzed separately to obtain estimates of direct heritability, maternal heritability, the proportion of phenotypic variance due to permanent environmental effects of the dam, and the correlation between direct genetic and maternal genetic effects. Traits analyzed included IGF-I at weaning, IGF28, IGF42, IGF56, mean IGF-I, birth weight, weaning weight, on-test weight, weight at day 28, 42, and 56 of the postweaning test, off-test weight, off-test hip height, and postweaning weight gain. The number of records available for analysis varied from 2056 for IGF42 to 2988 for birth weight, except that only 617 records were available for IGF-I at weaning.

The statistical model included the fixed effects of the birth year of the calf (1989 to 2017), the season of birth (spring vs. fall), IGF-I selection line (high vs. low), sex of calf (bull, heifer, or steer), and age of dam (2, 3, 4, 5–9, and ≥10 years. In addition, the breed of sire and breed of dam were included in the models as fixed effects for IGF-I concentration at weaning, birth weight, and weaning weight. All other traits were recorded only on purebred Angus calves. Weaning age was included as a linear covariate for weaning weight and IGF-I concentration at weaning. In contrast, the age of the calf at the beginning of the 140-day postweaning performance test was included as a linear covariate for postweaning traits.

Parameter estimates were first obtained for each trait using the full animal model. Three models (the full animal model and two reduced models) were then compared to determine which random effects should be included in the model for each trait in the pairwise bivariate analysis. All traits were analyzed using a full animal model that included fixed effects and random animal, maternal genetic, and permanent environmental effects. The analysis was then repeated using the two reduced models. In the first reduced model, the maternal permanent environmental effect was removed. The maternal permanent environmental and maternal genetic effects were removed in the second reduced model. These reduced models were compared to the full model using likelihood ratio tests. The most appropriate model was identified as the full or reduced model with a likelihood not significantly different from that of the full model. As a result, direct genetic and maternal genetic effects were included as random effects in the models for all traits other than IGF-I concentration at weaning, in which case the maternal genetic effect was not significant. The maternal permanent environmental effect of the dam was only significant and included in the models for the weight traits. The fixed and random effects included in the statistical models used to analyze each trait are summarized in Table 1. Parameter estimates for each trait were derived from the most appropriate model.

In all analyses, convergence was considered achieved when the variance of –2 log-likelihood from the simplex search algorithm was less than 10^−9^. After initial convergence, the process was restarted repeatedly until −2 log-likelihood values remained unchanged to the second decimal place from one restart to the next.

Next, IGF-I concentration at weaning, day 28, 42, and 56 of the postweaning test, and mean IGF-I concentration (trait 1) was paired with birth weight, weaning weight, on-test weight, weight at day 28, 42, and 56 of the postweaning performance test, off-test weight, off-test hip height, and postweaning gain (trait 2) in a series of bivariate analyses to estimate the additive genetic correlation between traits 1 and 2 (**r_A1A2_**), the correlation between the additive genetic effect for trait 1 and the maternal genetic effect for trait 2 (**r_A1M2_**), the correlation between the additive genetic effect for trait 2 and the maternal genetic effect for trait 1 (**r_A2M1_**), the environmental correlation between traits 1 and 2 (**r_E1E2_**), and the phenotypic correlation between the two traits (**r_P1P2_**). The MTDFREML programs allow for different models for each trait in a multiple-trait analysis. The same model was used for all traits in the bivariate analyses as in the single-trait analyses. In the multiple-trait analyses, convergence was assumed when the variance of −2 times the log-likelihood from the simplex search algorithm was less than 10^−9^, as in the single-trait analyses. The priors used in the multiple-trait analyses were the (co)variance solutions obtained from the single-trait analyses. To ensure that the log-likelihood was a global maximum and not a local one, “cold restarts” of the MTDFREML programs were performed using the converged values. Restarts continued until −2 times the log-likelihood remained unchanged to the second decimal place from one restart to the next.

The generation coefficient of each calf born in the selection experiment was determined using the method of Brinks et al. (1961), where the generation coefficient of the calf was equal to the average of the generation coefficients of the sire and dam plus one [13]. Foundation animals were assigned a generation coefficient of zero. The number of generations of selection was found by subtracting one from the average generation coefficient of the progeny.

## 3. Results

### 3.1. Descriptive Statistics and Genetic Parameter Estimates from Single-Trait Analyses

Means, standard deviations, coefficients of variation (CV), minimum values, and maximum values for growth traits and serum IGF-I concentrations are shown in Table 2. The coefficients of variation for the IGF-I measurements ranged from 50% to 70%, demonstrating the large variation present for this trait among the cattle in the selection experiment. The CVs for weight traits ranged from 14% to 21%, indicating moderate variation. Off-test hip heights were relatively uniform, with a CV of only 4%.

The average generation coefficient for calves born in 2007 was 5.71. Therefore, 4.71 generations of selection occurred (generation coefficient—1) before selection for IGF-I was relaxed.

Parameter estimates for serum IGF-I concentrations and performance traits are shown in Table 3. Direct heritability estimates (h_d_^2^) for IGF-I concentration ranged from 0.34 ± 0.07 for IGF56 to 0.46 ± 0.07 for mean IGF-I concentration. Direct heritability estimates for the weight traits were also moderate and varied from 0.32 ± 0.05 for birth weight to 0.39 ± 0.07 for off-test weight. In addition, postweaning weight gain and off-test hip height were moderately heritable (0.37 ± 0.07 and 0.49 ± 0.07, respectively).

The estimate of the heritability of maternal genetic effects (h_m_^2^) for IGF-I at weaning was 0.02 ± 0.11. Estimates varied from 0.10 ± 0.04 for IGF56 to 0.17 ± 0.05 for mean IGF-I, indicating only a small maternal genetic effect on IGF-I concentration. Maternal genetic heritability estimates were also small for the performance traits, ranging from 0.02 ± 0.03 for postweaning weight gain to 0.16 ± 0.05 for birth weight. The proportion of phenotypic variance accounted for by the maternal permanent environmental effects of the dam (c^2^) was near zero for all IGF-I measures. In terms of the weight traits, estimates of c^2^ were largest for weaning weight (0.22 ± 0.04) and declined throughout the postweaning period, reaching a low of 0.08 ± 0.03 for off-test weight. Estimates of c^2^ were near zero for postweaning weight gain and off-test hip height.

The estimated direct maternal correlation (r_am_) for IGF-I concentration at weaning was low but with a large standard error (−0.10 ± 0.94). The estimates of r_am_ for the other measures of IGF-I were large and negative (approximately −0.90). The estimate of r_am_ for birth weight was −0.33 ± 0.14 and ranged from −0.39 ± 0.29 for off-test weight to −0.61 ± 0.12 for weaning weight. Postweaning weight gain had an estimated r_am_ of −1.0 with a large standard error, whereas the estimated value for off-test hip height was −0.29 ± 0.21. Estimates of the direct maternal covariance (cov_am_) and the proportion of the phenotypic variance (σ_P_^2^) accounted for by this term are also shown in Table 3. Values for cov_am_/σ_P_^2^ were 0.01, 0.22, 0.23, 0.16, and 0.25 for IGF-I concentration at weaning, IGF28, IGF42, IGF56, and mean IGF-I, respectively. Therefore, the direct maternal covariance accounted for a relatively small proportion of the phenotypic variance even when the direct maternal correlation was large. Estimates of cov_am_/σ_P_^2^ were ≤0.14 for the performance traits.

### 3.2. Estimates of Genetic, Environmental, and Phenotypic Correlations Among IGF-I Concentrations at Weaning, Day 28, 42, and 56 of the Postweaning Test, and Mean IGF-I Concentration

Correlations among the IGF-I concentrations at different ages are shown in Table 4. Direct genetic correlations between serum IGF-I concentration at weaning and postweaning measures of IGF-I were large, ranging from 0.90 for IGF28 to 0.63 for IGF56. The direct genetic correlations declined in magnitude as the time between weaning and the postweaning IGF-I measures increased. The correlation between IGF-I at weaning and the mean postweaning concentration was 0.69. Direct genetic correlations among IGF28, IGF42, IGF56, and mean IGF-I were all large and positive (≥0.89).

Estimates of the correlations between the additive genetic effect for IGF-I at weaning and the maternal genetic effects for the other measures of IGF-I (r_A1M2_) ranged from −0.32 to −0.64. Estimates of r_A1M2_ for IGF28, IGF42, IGF56, and mean IGF-I were large and negative (≥−0.78) except for the near-zero correlation between IGF28 and IGF42. Likewise, estimates of the correlations between the maternal genetic effects for postweaning IGF-I concentration at one time point and the additive genetic effect for postweaning IGF-I concentration at a different time point (r_A2M1_) were large and negative (≥−0.51).

Correlations between the additive genetic effects and maternal genetic effects for the same IGF-I measurement (r_A1M1_ and r_A2M2_) were ≥−0.86 except when IGF28 was paired with IGF42, in which case the estimated correlations were of moderate magnitude. Maternal genetic effects were highly correlated (r_M1M2_) for all combinations of IGF-I measurements (≥0.93 except for IGF42 and IGF56, in which case the correlation was 0.74).

Environmental correlations (r_E1E2_) between IGF-I at weaning and the postweaning measures of IGF-I varied between 0.26 and 0.38. In contrast, the estimates ranged from 0.45 to 0.82 between the environmental effects for IGF28, IGF42, IGF56, and mean IGF-I. Estimates of the phenotypic correlations (r_P1P2_) were moderate to large, ranging from 0.37 between IGF-I at weaning and IGF56 to 0.89 between IGF42 and mean IGF-I.

### 3.3. Estimates of Genetic, Environmental, and Phenotypic Correlations of IGF-I Measurements at Weaning, Day 28, 42, and 56 of the Postweaning Performance Test and Mean IGF-I Concentration with Weight Traits

Estimates of the genetic, environmental, and phenotypic correlations of IGF-I measurements taken at various ages with weight traits are shown in Table 5. The direct genetic correlations of the IGF-I measurements with birth weight were negative and ranged from −0.08 ± 0.14 to −0.29 ± 0.14. The direct genetic correlation of IGF-I at weaning with weaning weight was negative but small with a large standard error (r_A1A2_ = −0.12 ± 0.33). In contrast, the correlations of postweaning IGF-I measures with weaning weight were positive but small, ranging from 0.11 ± 0.12 to 0.19 ± 0.14. The additive genetic correlation of IGF-I at weaning with on-test weight was −0.12, and the correlations of IGF-I concentration at the other time points with on-test weight varied from 0.04 ± 0.13 to 0.19 ± 0.14. IGF-I at weaning exhibited low correlations with weights at days 28, 42, and 56 of the postweaning test and with off-test weight (−0.09 ≤ r_A1A2_ ≤ 0.14). Estimates of r_A1A2_ ranged from 0.08 ± 0.13 to 0.26 ± 0.14 when IGF28, IGF42, IGF56, and mean IGF-I were paired with weights at days 28, 42, and 56 of the postweaning test and with off-test weight.

The direct maternal correlation was consistently large and negative for all postweaning measures of IGF-I (−0.88 ≤ r_A1M1_ ≤ 0.91) regardless of which weight trait was paired with the IGF-I measurement. The direct maternal correlation for birth weight (r_A2M2)_ ranged from −0.30 ± 0.15 to −0.36 ± 0.44 depending on the IGF-I measurement paired with birth weight. For weaning weight, estimates of r_A2M2_ were negative and moderate to large (−0.38 ± 0.29 ≤ r_A2M2_ ≤ −0.60 ± 0.13). When paired with IGF-I at weaning, estimates of r_A2M2_ for the postweaning weights ranged from −0.18 to −0.42, whereas when paired with IGF28, IGF42, IGF56, or mean IGF-I, these estimates were between −0.32 ± 0.28 and −0.60 ± 0.16.

The correlation between the additive genetic effects of IGF-I at weaning and the maternal genetic effects of the weight traits (r_A1M2_) were large and positive, with a minimum estimate of 0.57 for birth weight and a maximum estimate of 0.95 for off-test weight. Estimates of r_A1M2_ were moderate when trait 1 was one of the postweaning measures of IGF-I and trait 2 was birth weight (0.31 ± 0.16 to 0.48 ± 0.16). Estimates of r_A1M2_ were low to moderate when trait 1 was one of the postweaning measures of IGF-I and trait 2 was weaning weight or a postweaning weight (−0.11 ± 0.22 to 0.27 ± 0.41). Likewise, estimates of the correlation between the additive genetic effects for trait 2 (a weight trait) and the maternal genetic effects for trait 1 (an IGF-I trait) were low to moderate (−0.30 ± 0.17 to 0.10 ± 0.17) and, in most cases, were small and negative.

Correlations between the maternal effects of the IGF-I traits and birth weight (r_M1M2_) ranged from −0.25 ± 0.17 to −0.38 ± 0.20. For the other trait combinations, the estimates were generally positive and varied between −0.04 ± 0.23 and 0.28 ± 0.41. The environmental correlations between the IGF-I traits and birth weight and between the postweaning IGF-I measurements and weaning weight were near zero. Estimates of the environmental correlations (r_E1E2_) of IGF-I at weaning with weaning weight, on-test weight, and weight at days 28, 42, and 56 of the postweaning period ranged from 0.56 to 0.76. In contrast, the environmental correlation of IGF-I at weaning with off-test weight was 0.35. Estimates of r_E1E2_ varied between 0.04 ± 0.05 and 0.22 ± 0.06.

Finally, the phenotypic correlation (r_P1P2_) of IGF-I at weaning with birth weight was −0.06, and the other weight traits varied from 0.35 to 0.49. Estimates of the phenotypic correlations between postweaning IGF-I concentrations and birth and weaning weights were low (−0.08 ≤ r_P1P2_ ≤ 0.09). The phenotypic correlations between postweaning IGF-I and postweaning weights ranged from 0.08 to 0.19.

### 3.4. Estimates of Genetic, Environmental, and Phenotypic Correlations of IGF-I Concentrations at Weaning, Day 28, 42, and 56 of the Postweaning Test and Mean IGF-I Concentration with Postweaning Weight Gain and Off-Test Hip Height

Estimates of the additive genetic correlations (r_A1A2_) between IGF-I measurements and postweaning weight gain and off-test hip height were positive and low to moderate in magnitude (0.12 to 0.45; Table 6). The correlations between the additive direct and maternal genetic effects within the same trait (r_A1M1_ and r_A2M2_) for IGF-I measurements and postweaning gain were large and negative (−0.89 ± 0.06 to −0.92 ± 0.04 and −0.86 to −0.96 ± 0.30, respectively). These large negative estimates for r_A1M1_ persisted when IGF-I concentrations were paired with off-test hip height (−0.87 ± 0.06 to −0.90 ± 0.04). However, in the bivariate analysis, the direct maternal correlations for off-test hip height were moderate, ranging from −0.20 ± 0.16 to −0.27. Estimates of r_A1M2_ and r_A2M1_ were generally low to moderate, ranging from −0.27 ± 0.37 to 0.23 ± 0.23, except for a notable correlation of 0.87 between the additive genetic effect for IGF-I at weaning and the maternal genetic effect for off-test hip height.

Estimates of the correlations between the maternal effects of the two traits (r_M1M2_) in the bivariate analyses varied based on the IGF measurement used. The correlations were nearly zero when IGF28 was paired with postweaning gain and off-test hip height. When IGF42 was paired with these traits, the correlations were positive but low. With IGF56, the correlations were negative and moderate in magnitude. Finally, the correlations were negative but small when mean IGF-I was used.

Environmental correlations between IGF-I measurements and postweaning weight gain and off-test hip height (r_E1E2_) were small, ranging from 0.00 ± 0.06 to 0.19. Similarly, small phenotypic correlations were observed between IGF-I concentrations and the two postweaning traits (r_P1P2_), varying between 0.12 and 0.15, except for a higher correlation of 0.28 between IGF-I at weaning and off-test hip height.

## 4. Discussion

### 4.1. Descriptive Statistics

The large ranges, SD, and CV for serum IGF-I concentration (Table 2) indicate the wide variation present for this trait among the cattle included in this study. Other authors [14,15,16,17] have also observed large variations in circulating IGF-I concentration. The CVs for weight traits ranged from 14% to 21%, indicating moderate variation. The CV for weight traits such as birth weight, weaning weight, and yearling weight in beef cattle typically ranges from 10% to 20%. Off-test hip heights were relatively uniform, with a CV of only 4%.

### 4.2. Genetic Parameter Estimates from Single-Trait Analyses

Direct heritability estimates for serum IGF-I concentration ranged from 0.34 ± 0.07 for IGF56 to 0.46 ± 0.07 for mean IGF-I concentration (Table 3), indicating serum IGF-I concentration is moderately heritable and expected to respond to selection. These estimates are consistent with those of Davis and Simmen [18], who used data from earlier years (1989–2000) of the same selection experiment and analyzed 1761 records for mean IGF-I. The current study, which includes data from 1989 through to 2017, involved 2180 records for mean IGF-I and 617 records for IGF-I concentration at weaning. The estimates from the current study are slightly larger than the values of 0.31 ± 0.18 observed at birth [19], 0.32 ± 0.06 at 8 to 10 months of age [14], 0.34 ± 0.09 and 0.43 ± 0.12 at 9 and 22 months of age, respectively, in cattle fed at two different locations in Australia [15], 0.36 ± 0.09 at weaning (mean age = 237 days) in seven Australian seedstock herds [10], and 0.32 ± 0.06 at weaning (mean age = 201 days) and 0.30 ± 0.07 during the postweaning period (average age = 310 days) in Angus seedstock cattle fed on pasture in Australia [16]. Direct heritability estimates for the weight traits were also in the moderate range and varied from 0.32 ± 0.05 for birth weight to 0.39 ± 0.07 for off-test weight. In addition, postweaning weight gain and off-test hip height were moderately heritable (0.37 ± 0.07 and 0.49 ± 0.07, respectively). The direct heritability estimates of body weight and weight gain generally agree with average estimates from many studies, as summarized in [20,21,22]. These findings suggest that a significant genetic component influences weight, weight gain, height, and serum IGF-I concentration, which could be effectively utilized in breeding programs designed to increase the growth and IGF-I concentrations of beef cattle.

The heritability estimates for maternal genetic effects (h_m_^2^) for IGF-I concentrations and performance traits in this study provide insightful implications about the influence of maternal genetics on these traits. Specifically, the heritability of maternal genetic effects for IGF-I concentration at weaning was near zero (0.02 ± 0.11), suggesting a minimal maternal genetic influence. Although there was some variation, with estimates ranging from 0.10 ± 0.04 for IGF56 to 0.17 ± 0.05 for mean IGF-I, these values collectively indicate that maternal genetic effects on serum IGF-I concentration in cattle are small. These estimates agree well with those based on data from earlier years of this selection experiment [23].

Maternal genetic heritability estimates for the performance traits were also low, ranging from 0.02 ± 0.03 for postweaning weight gain to 0.16 ± 0.05 for birth weight. These findings underscore that maternal genetic contributions to these traits are limited. This trend aligns with previous research suggesting that direct genetic effects are more prominent than maternal genetic effects in influencing growth traits. Low maternal heritabilities have been previously reported for birth, weaning, and yearling weight [21,22].

In addition, the proportion of phenotypic variance due to maternal permanent environmental effects (c^2^) was near zero for all IGF-I measures, indicating that these effects do not significantly contribute to the variability in circulating IGF-I. The maternal permanent environmental effects were variable for the weight traits. Estimates of c^2^ were highest for weaning weight (0.22 ± 0.04), suggesting a notable maternal environmental influence at this early stage of life when the calves are nursing their mothers. However, these effects decreased throughout the postweaning period, reaching a low of 0.08 ± 0.03 for off-test weight. The c^2^ estimates were near zero for postweaning weight gain and off-test hip height, illustrating the maternal environment’s diminishing influence following weaning. In his review, Mohiuddin [21] found that estimates of c^2^ averaged 0.03, 0.07, and 0.03 for birth, weaning, and yearling weight, respectively.

These findings highlight the predominant role of direct genetic and postnatal environmental factors in determining the phenotypic expression of IGF-I and performance traits. The lack of maternal genetic and permanent environmental effects suggests that genetic selection strategies focused on direct genetic effects rather than maternal components may be more effective in improving these traits.

Willham [24] stated that knowing the sign and magnitude of the correlation between direct and maternal effects of traits with high economic importance is critical to designing optimal breeding plans for most domestic animals. Estimates of direct maternal correlations (r_am_) for serum IGF-I concentrations and performance traits reveal important insights into the interplay between direct genetic effects and maternal influences for these traits. The estimate of r_am_ for IGF-I concentration at weaning was low (−0.10) with a large standard error (±0.94), indicating considerable uncertainty in this estimate. For other measures of IGF-I, the r_am_ estimates were large and negative (approximately −0.90), suggesting a strong antagonistic relationship between direct and maternal genetic effects for serum IGF-I concentration.

Estimates of r_am_ were variable for the growth traits. The estimate for birth weight was moderately negative (−0.33 ± 0.14), whereas for other weight traits, the values ranged from −0.39 ± 0.29 for off-test weight to −0.61 ± 0.12 for weaning weight. The estimate of r_am_ for postweaning weight gain was −1.0, likely due to a problem with convergence. As described in the Materials and Methods section, in all analyses, convergence was considered achieved when the variance of –2 log-likelihood from the simplex search algorithm was less than 10^−9^. After initial convergence, the process was restarted repeatedly until −2 log-likelihood values remained unchanged to the second decimal place from one restart to the next.

The r_am_ estimate for off-test hip height was negative (−0.29 ± 0.21), though of smaller magnitude compared to the other traits. In the studies he summarized, Mohiuddin [21] reported that r_am_ averaged −0.35, −0.15, and −0.26 for birth, weaning, and yearling weight. Koots et al. [25] found that r_am_ averaged −0.27 for birth weight and −0.30 for weaning weight. Robinson [26] reported that estimates of direct maternal genetic correlations were large and negative for Australian Angus cattle. Robinson [26] further commented that estimates of genetic correlations between additive direct and maternal effects in beef cattle vary widely, and most estimates tend to be negative in Australian, American, and Argentine cattle. Negative correlations between direct and maternal genetic effects may be due not only to genetic antagonisms but also to negative environmental dam–offspring covariances or sire-by-year variation that is unaccounted for [27].

Despite the large direct maternal correlations, the proportion of phenotypic variance explained by the direct maternal covariance (cov_am_/σ_p_^2^) for IGF-I measures was relatively small. The values were 0.01, 0.22, 0.23, 0.16, and 0.25 for IGF-I concentration at weaning, IGF28, IGF42, IGF56, and mean IGF-I, respectively. These findings suggest that, while the direct maternal correlations are strong, the impact of these covariances on the overall phenotypic variance is limited. The cov_am_/σ_p_^2^ values were all ≤0.14 for the growth traits, indicating that the direct maternal covariance accounted for an even smaller proportion of the phenotypic variance in these traits. This implies that other factors, including direct genetic effects and environmental influences, play a larger role in determining the phenotypic expression of performance traits in beef cattle.

### 4.3. Genetic, Environmental, and Phenotypic Correlations Among Serum IGF-I Concentrations at Different Time Points

The correlations among serum IGF-I concentrations at different ages provide valuable insights into the genetic relationships among these measures. Direct genetic correlations between IGF-I concentration at weaning and postweaning measures of IGF-I were consistently large. However, they declined as the interval between the weaning and postweaning measurements increased. Specifically, the direct genetic correlation between IGF-I at weaning and IGF28 was very large (0.90), but it decreased to 0.63 for IGF56. The correlation between IGF-I at weaning and the mean postweaning concentration was also strong (0.69). Moore et al. [16] reported a genetic correlation of 1.0 ± 0.04 between IGF-I concentration measured at weaning (mean age = 201 days) and during the postweaning period (mean age = 310 days) in Australian Angus seedstock cattle fed on pasture. These results demonstrate that genetic factors influencing IGF-I concentrations at weaning continue to substantially affect IGF-I concentrations during the postweaning period, although the influence diminishes over time. The large correlations indicate that selecting for higher IGF-I concentration at weaning is expected to increase IGF-I concentration later in life, albeit with a slightly reduced impact as animals age.

Furthermore, the direct genetic correlations among the postweaning IGF-I measures (IGF28, IGF42, IGF56) and the mean IGF-I concentration were all large and positive, with values of 0.89 or greater. These strong correlations imply a consistent genetic basis for IGF-I concentrations throughout the postweaning period. This consistency suggests that genetic selection for IGF-I concentration at any postweaning age will likely yield increases across all postweaning time points.

The estimates of correlations between the additive genetic effect for IGF-I at weaning and the maternal genetic effects for postweaning IGF-I measures (r_A1M2_) ranged from −0.32 to −0.64. Notably, the correlations for IGF28, IGF42, IGF56, and mean IGF-I were large and negative (≥−0.78), except for the near-zero correlation between IGF28 and IGF42. These negative correlations suggest a strong antagonistic relationship between the direct genetic effects at weaning and the maternal genetic effects influencing postweaning IGF-I concentrations. Similarly, the correlations between the maternal genetic effects for postweaning IGF-I at one time point and the additive genetic effect for IGF-I at another postweaning time point (r_A2M1_) were large and negative (≥−0.51). This indicates a consistent antagonistic interaction between direct and maternal genetic influences across different postweaning periods, complicating the genetic improvement of these traits due to the opposing directions of the genetic effects. Likewise, the correlations between the additive genetic effects and maternal genetic effects for the same IGF-I measurement (r_A1M1_ and r_A2M2_) were generally large and negative (≥−0.86), except when IGF28 was paired with IGF42, where the correlations were of moderate magnitude. These large negative correlations agree with those from the single-trait analyses (Table 3) and suggest a strong negative relationship between direct and maternal genetic effects when measured at the same time point.

Moreover, maternal genetic effects were highly correlated (r_M1M2_) across all combinations of IGF-I measurements, with values ≥0.93, except for IGF42 and IGF56, which had a correlation of 0.74. This high degree of correlation among maternal effects implies a consistent maternal influence across different ages, suggesting that maternal genetic improvement in one time period is likely to be beneficial across other periods as well.

Environmental correlations (r_E1E2_) between IGF-I at weaning and postweaning measures ranged from 0.26 to 0.38, indicating moderate shared environmental influences between these time points. The environmental correlations among postweaning IGF-I measures (IGF28, IGF42, IGF56, and mean IGF-I) were even stronger, ranging from 0.45 to 0.82. These results suggest that environmental factors affecting IGF-I concentrations are more consistent during the postweaning period than between the weaning and postweaning stages.

Phenotypic correlations (r_P1P2_) were moderate to large, with values ranging from 0.37 between IGF-I at weaning and IGF56 to 0.89 between IGF42 and mean IGF-I. These high phenotypic correlations demonstrate that animals with higher IGF-I concentrations at one age are likely to maintain higher concentrations at other ages, reflecting the combined effects of genetics and environment.

The results highlight the intricate interplay between direct and maternal genetic effects on IGF-I concentrations across different ages. The strong antagonistic correlations between direct and maternal genetic effects for different time points present a challenge for simultaneously improving these traits. However, the large correlations among maternal effects provide opportunities for targeted selection strategies. Understanding these dynamics is crucial for developing effective breeding programs to optimize IGF-I concentrations. The genetic, environmental, and phenotypic correlations of the IGF-I measurements taken on days 28, 42, and 56 of the postweaning period with mean IGF-I concentration were large, as expected, due to the part–whole relationship between a mean and its components and agreed closely with the correlations presented by Davis and Simmen [17], who analyzed data from the earlier years of the same selection experiment. Given the large genetic correlations observed in the current study and in the literature [16,18], as well as the magnitude of the heritability of IGF-I (Table 3), a single postweaning measurement of serum IGF-I concentration should be sufficient for selection purposes, rather than using the mean of multiple measurements for selection. Alternatively, selection based on IGF-I concentration at weaning is also expected to be effective, given its moderate heritability and large genetic correlations with postweaning IGF-I concentrations.

### 4.4. Genetic, Environmental, and Phenotypic Correlations of IGF-I Concentrations at Various Time Points with Weights at Different Ages

This study aimed to estimate the genetic, environmental, and phenotypic correlations of IGF-I measurements taken at various ages with weight traits in livestock. The results presented in Table 5 reveal several key insights into the relationships between these traits.

The direct genetic correlations of IGF-I measurements with birth weight were negative, ranging from −0.08 ± 0.14 to −0.29 ± 0.14. This indicates that higher IGF-I concentrations are genetically associated with lower birth weights, although the correlations are relatively weak. Similarly, the direct genetic correlation of IGF-I at weaning with weaning weight and the postweaning weights was small and variable (−0.12 to 0.14), suggesting a minimal genetic influence of IGF-I at weaning on the weights of beef cattle. Postweaning IGF-I measures showed positive but small genetic correlations with weaning weight (0.11 ± 0.12 to 0.19 ± 0.14), indicating a weak genetic link between postweaning IGF-I concentrations and heavier weights at weaning. Likewise, the estimates of r_A1A2_ when IGF-I at different postweaning stages was paired with postweaning weights ranged from 0.04 ± 0.13 to 0.26 ± 0.14. The genetic correlations generally increased from day 0 to day 140 of the postweaning test. Estimates of genetic correlations between concentrations of circulating IGF-I and body weights at different ages have also varied in the literature. Johnston et al. [14] found a genetic correlation of 0.11 ± 0.14 between IGF-I and postweaning live weight. On the other hand, Johnston et al. [15] observed genetic correlations of −0.25 ± 0.25 and 0.03 ± 0.14 between IGF-I concentrations and mid-test weight in groups of cattle fed at two locations in Australia. Phenotypic correlations of weaning IGF-I with birth weight, 200-day weight, and pre-weaning ADG in seven Angus seedstock herds in Australia were near zero [10]. In addition, the same authors reported that plasma IGF-I concentration at weaning was not genetically correlated with birth weight but had genetic correlations of −0.40 ± 0.17 and −0.52 ± 0.16, respectively, with 200-day weight and ADG. Moore et al. [16] reported direct genetic correlations of IGF-I with birth weight, 200-day weight, and 400-day weight of −0.22 ± 0.08, −0.17 ± 0.09, and −0.10 ± 0.14, respectively.

In the bivariate analyses (Table 5), the direct maternal correlations were consistently large and negative for all postweaning IGF-I measures (−0.88 ≤ r_A1M1_ ≤ −0.91), indicating a strong antagonistic relationship between the direct and maternal genetic effects. These negative correlations were in close agreement with the results of the single-trait analyses (Table 3).

When considering the correlation between the additive genetic effects of IGF-I at weaning and the maternal genetic effects of the weight traits (r_A1M2_), the estimates were large and positive for birth weight (minimum estimate of 0.57) and off-test weight (maximum estimate of 0.95). For postweaning measures of IGF-I paired with birth weight, the estimates were moderate (0.31 ± 0.16 to 0.48 ± 0.16), while estimates were low to moderate when postweaning IGF-I measures were paired with weaning weight or postweaning weights (−0.11 ± 0.22 to 0.27 ± 0.41). These correlations indicate a complex interplay between direct and maternal genetic effects across different growth stages. The correlations between the additive genetic effects for weight traits and the maternal genetic effects for IGF-I traits (r_A2M1_) were generally low to moderate (−0.30 ± 0.17 to 0.10 ± 0.17) and were mostly small and negative. This suggests limited and inconsistent maternal genetic influence of IGF-I on weight traits. Estimates of such correlations reported in the literature have also been inconsistent. Correlations between the direct component of IGF-I and the maternal component of birth weight and 200-day weight were 0.15 ± 0.13 and 0.31 ± 0.11, respectively [16]. The authors suggested that selection for reduced IGF-I concentration may result in decreased maternal weight. On the other hand, Herd et al. [28] found evidence of a negative relationship between plasma IGF-I concentration at the conclusion of a postweaning test and the maternal component of birth weight and weaning weight, indicating that selection for lower IGF-I concentration should lead to improved maternal nutrition to the calf before and after birth.

Maternal effects correlations (r_M1M2_) between IGF-I traits and birth weight ranged from −0.25 ± 0.17 to −0.38 ± 0.20. The estimates were generally positive for other trait combinations but varied widely (−0.04 ± 0.23 to 0.28 ± 0.41). This variability underscores the complexity of maternal effects on IGF-I and weight traits.

Environmental correlations (r_E1E2_) between IGF-I traits and birth weight and postweaning IGF-I measurements with weaning weight were near zero, indicating minimal shared environmental influences. However, IGF-I at weaning exhibited large environmental correlations with weaning weight and postweaning weights (0.56 to 0.76) other than off-test weight (0.35), highlighting a notable environmental impact of IGF-I concentration at weaning on weight traits during specific growth phases. Estimates of r_E1E2_ involving postweaning measures of IGF-I and postweaning weight traits were small to moderate (≤0.22). Therefore, a tendency existed for environmental improvements to result in increased serum IGF-I concentrations and increased body weight. Numerous studies have shown that diet, including energy and protein content, influences plasma and serum IGF-I concentrations in cattle [29,30,31,32]. Undernutrition can attenuate the IGF-I response to growth hormone and uncouple the regulation of IGF-I commonly ascribed to growth hormone [32]. Indeed, the concentration of circulating IGF-I can be used as an objective indicator of nutritional status in beef cattle [33,34].

Lastly, the phenotypic correlations (r_P1P2_) of IGF-I at weaning with birth weight were low (−0.06) but ranged from 0.35 to 0.49 for the other weight traits. Phenotypic correlations between postweaning IGF-I concentrations and birth and weaning weights were low (−0.08 ≤ r_P1P2_ ≤ 0.09), while those with postweaning weights ranged from 0.08 to 0.19. These findings suggest that the observable influence of IGF-I on weight traits is modest but more pronounced in the postweaning period. Moore et al. [16] reported phenotypic correlations between the direct component of IGF-I and the direct components of birth weight, 200-day weight, and 400-day weight of −0.10, 0.06, and 0.16, respectively, which agree well with the phenotypic correlations from the current study.

In summary, our study’s results demonstrate the intricate genetic, environmental, and phenotypic relationships between IGF-I concentrations and weight traits across different growth stages, with significant implications for understanding and potentially managing growth performance in beef cattle.

### 4.5. Genetic, Environmental, and Phenotypic Correlations of IGF-I Concentrations at Various Time Points with Postweaning Weight Gain and Off-Test Hip Height

The additive genetic correlations (r_A1A2_) between IGF-I concentration at weaning and postweaning weight gain and off-test hip height were 0.45 and 0.24, respectively. The correlations involving postweaning IGF-I concentrations were positive and ranged from low to moderate (0.12 to 0.32; Table 6). This suggests a modest genetic association between higher IGF-I concentrations and improved growth performance as measured by postweaning weight gain and hip height. It is interesting to note that IGF-I at weaning is a better indicator of postweaning weight gain than are postweaning measures of IGF-I. In contrast to the positive correlations found in the current study, Johnston et al. [14] reported a negative genetic correlation of −0.25 ± 0.20 between IGF-I concentration and finishing average daily gain in temperate cattle breeds in Australia. In addition, Johnston et al. [15] obtained genetic correlation estimates of −0.23 ± 0.32 and −0.20 ± 0.17 between IGF-I concentration and average daily gain during postweaning tests involving two groups of Australian cattle. Herd et al. [28] also found evidence of a negative relationship between plasma IGF-I concentration at the end of a 70-day postweaning test and average daily gain during the test. Estimates of genetic correlations between IGF-I concentration and average daily weight gain have also varied in sign and magnitude in pigs [17], possibly due to sampling effects or due to the growth phase (periods of lean vs. fat accretion) during which the performance tests occurred [35].

Notably, the correlations between the additive direct and maternal genetic effects within the same trait (r_A1M1_ and r_A2M2_) for IGF-I measurements and postweaning gain were large and negative, ranging from −0.89 to −0.92 and −0.86 to −0.96, respectively. These strong negative correlations indicate substantial opposing influences of direct and maternal genetic effects on these traits. This trend persisted for IGF-I concentrations paired with off-test hip height, with r_A1M1_ estimates ranging from −0.87 ± 0.06 to −0.90 ± 0.04. These results were consistent with those observed in the single-trait analysis (Table 3). In contrast, in the bivariate analysis, the direct maternal correlations for off-test hip height were moderate, ranging from −0.20 to −0.27. These moderate correlations suggest a lesser, though still notable, degree of opposition between direct and maternal genetic effects for hip height compared to IGF-I measurements and weight gain following weaning.

The correlations between the maternal genetic effects (r_M1M2_) of the two traits in the bivariate analyses varied depending on the IGF measurement. When IGF28 was paired with postweaning gain and off-test hip height, the correlations were nearly zero, indicating minimal shared maternal influence. However, when IGF42 was paired with these traits, the correlations were positive but low, suggesting a small shared maternal genetic component. With IGF56, the correlations were negative and moderate, indicating a more substantial yet opposing maternal influence. Finally, when mean IGF-I was used, the correlations were negative but small, highlighting a slight opposing maternal influence across these traits.

The environmental correlations (r_E1E2_) of IGF-I measurements with postweaning gain and off-test hip height were small, ranging from 0.00 to 0.19. This indicates the shared environmental influences on IGF-I concentrations and these growth traits are minimal, suggesting that the environmental factors affecting IGF-I concentrations are largely independent of those affecting postweaning weight gain and hip height.

The phenotypic correlations (r_P1P2_) between IGF-I concentrations and the two postweaning traits were small, generally varying only from 0.12 to 0.15. These low phenotypic correlations suggest a modest relationship between IGF-I concentrations and growth traits in beef cattle, in contrast to rodents, and may be attributed to several factors. First, given that growth hormone is the primary physiological determinant of growth rate, other intermediaries of growth hormone action in muscle tissue, such as insulin-like growth factor-binding protein-3 (IGFBP3), may exert species-specific control of IGF-I bioavailability [36]. Second, the persistent expression of family member IGF-II, a known growth mediator, rather than of IGF-I occurs during the postnatal stages of domestic species but not in rodents [37]. Third, bioactive IGF-I, rather than serum concentrations, may be more predictive of growth phenotype [38].

Govoni et al. (2003) found small but significant simple correlations between IGF-I concentrations measured weekly and average daily gain from birth to weaning in male (r = 0.21; *p* < 0.001) and female (r = 0.12; *p* < 0.05) Hereford calves [39]. We observed a larger correlation (0.28) between IGF-I at weaning and off-test hip height. This result suggests that IGF-I concentration at weaning may have a more noticeable impact on hip height at the end of the postweaning period, potentially reflecting a specific developmental phase where IGF-I plays a more critical role.

In summary, the small to moderate additive genetic, environmental, and phenotypic correlations observed between IGF-I measurements and key growth traits underscore the complex interplay of factors influencing growth performance in livestock. These findings suggest that while IGF-I concentration has some influence, it is not the primary determinant of weight gain or hip height. Therefore, genetic selection strategies should consider the broader genetic architecture and environmental factors affecting these traits to optimize growth performance.

## 5. Conclusions

This study investigated the genetic, environmental, and phenotypic relationships among serum IGF-I concentrations and various growth traits in beef cattle, offering insights into breeding programs and genetic selection strategies. The direct heritability of serum IGF-I concentration ranged from 0.34 to 0.46, suggesting moderate heritability and potential for selection, consistent with previous studies. Maternal genetic effects for IGF-I concentrations were minimal, and maternal permanent environmental effects were negligible for IGF-I but more relevant for weight traits, particularly weaning weight. Strong negative direct maternal correlations for IGF-I concentrations and weight traits indicate an antagonistic relationship, posing challenges for genetic improvement.

Large genetic correlations among serum IGF-I concentrations across ages suggest that selection at one age will influence IGF-I concentrations throughout the growth phase. The genetic correlations between IGF-I concentrations and weight traits varied, indicating a need for balanced selection strategies. Environmental and phenotypic correlations between IGF-I and growth traits were generally low, suggesting minimal shared environmental influences.

Our study highlights the moderate heritability of serum IGF-I concentrations and growth traits, the limited role of maternal genetic effects, and the complexity of genetic correlations. These findings suggest that genetic selection can enhance these traits but requires a holistic approach that considers multiple traits. Future research should further explore these dynamics to optimize breeding programs and improve the growth performance of beef cattle.

## Figures and Tables

**Table 1:** Fixed and random effects for traits included in the variance component analysis.

				Fixed Effects						Random Effects	
Dependent Variable	Season	Line	Age	Birth Year	Sex	Sire Breed	Dam Breed	Age of Dam	Direct Genetic	Maternal Genetic	Maternal Permanent Environment
IGF Wean	X	X	X	X	X	X	X	X	X		
IGF28 ^a^	X	X	X	X	X			X	X	X	
IGF42	X	X	X	X	X			X	X	X	
IGF56	X	X	X	X	X			X	X	X	
Mean IGF-I ^b^	X	X	X	X	X			X	X	X	
Birth wt	X	X		X	X	X	X	X	X	X	X
Weaning wt	X	X	X	X	X	X	X	X	X	X	X
On-test wt	X	X	X	X	X			X	X	X	X
day-28 wt	X	X	X	X	X			X	X	X	X
day-42 wt	X	X	X	X	X			X	X	X	X
day-56 wt	X	X	X	X	X			X	X	X	X
Off-test wt	X	X	X	X	X			X	X	X	X
Postweaning weight gain	X	X	X	X	X			X	X	X	
Off-test hip ht	X	X	X	X	X			X	X	X	

^a^ IGF28, IGF42, and IGF56 are the IGF-I concentrations on days 28, 42, and 56 of the 140-day postweaning test. ^b^ Mean IGF-I is the average of serum IGF-I measurements taken at days 28, 42, and 56 of the postweaning period for each calf. An X in the table indicates that a given effect was included in the statistical model used to analyze a dependent variable.

**Table 2:** Means, standard deviations, and coefficients of variation (CV) for serum IGF-I concentrations and performance traits.

Trait	N	Mean	SD	CV, %	Minimum Value	Maximum Value
IGF Wean, ng/mL	617	198	98	50	33	538
IGF28 ^a^, ng/mL	2123	239	166	70	4	1026
IGF42, ng/mL	2056	263	177	67	15	1031
IGF56, ng/mL	2126	256	176	69	3	975
Mean IGF-I ^b^, ng/mL	2180	245	166	68	4	913
Birth wt, kg	2988	34	5	14	11	57
Weaning wt, kg	2598	200	31	16	91	299
On-test wt, kg	2191	223	37	16	84	347
day-28 wt, kg	2186	258	45	18	98	408
day-42 wt, kg	2057	276	50	18	93	438
day-56 wt, kg	2184	292	55	19	102	460
Off-test wt, kg	2138	388	83	21	147	608
Postweaning gain, kg	2138	165	62	38	18	330
Off-test hip ht, cm	2138	116	5	4	99	132
Weaning age, days	2598	217	27	13		276
On-test age, days	2191	252	21	8		307

^a^ IGF28, IGF42, and IGF56 are the IGF-I concentrations on days 28, 42, and 56 of the 140-day postweaning test. ^b^ Mean IGF-I is the average of serum IGF-I measurements taken at days 28, 42, and 56 of the postweaning period for each calf.

**Table 3:** Parameter estimates ^a^ for serum IGF-I concentrations and performance traits derived from single-trait full animal model.

Trait	σ_p_^2^	h_d_^2^	h_m_^2^	c^2^	r_am_	cov_am_	cov_am_/σ_p_^2^
IGF Wean	4653	0.42 ± 0.14	0.02 ± 0.11	0.01 ± 0.06	−0.10 ± 0.94	−41.9	0.01
IGF28 ^b^	11,403	0.41 ± 0.07	0.15 ± 0.04	0.00 ± 0.02	−0.91 ± 0.06	−2557	0.22
IGF42	13,273	0.42 ± 0.07	0.16 ± 0.05	0.00 ± 0.02	−0.90 ± 0.06	−3096	0.23
IGF56	11,898	0.34 ± 0.07	0.10 ± 0.04	0.00 ± 0.02	−0.88 ± 0.08	−1873	0.16
Mean IGF-I ^c^	9076	0.46 ± 0.07	0.17 ± 0.05	0.00 ± 0.02	−0.90 ± 0.05	−2308	0.25
Birth wt	18	0.32 ± 0.05	0.16 ± 0.05	0.03 ± 0.03	−0.33 ± 0.14	−1.36	0.07
Weaning wt	676	0.38 ± 0.06	0.15 ± 0.05	0.22 ± 0.04	−0.61 ± 0.12	−97.9	0.14
On-test wt	884	0.33 ± 0.06	0.10 ± 0.05	0.19 ± 0.04	−0.60 ± 0.15	−99.4	0.11
day-28 wt	1098	0.37 ± 0.06	0.09 ± 0.05	0.15 ± 0.03	−0.52 ± 0.16	−105.9	0.10
day-42 wt	1173	0.36 ± 0.06	0.08 ± 0.05	0.14 ± 0.03	−0.46 ± 0.19	−92.6	0.08
day-56 wt	1266	0.36 ± 0.06	0.09 ± 0.04	0.12 ± 0.03	−0.49 ± 0.17	−112.2	0.09
Off-test wt	1777	0.39 ± 0.07	0.03 ± 0.03	0.08 ± 0.03	−0.39 ± 0.29	−71.6	0.04
Postweaning gain	3002	0.37 ± 0.07	0.02 ± 0.03	0.00 ± 0.02	−1.0 ± 0.61	−230.1	0.08
Off-test hip ht	15	0.49 ± 0.07	0.05 ± 0.03	0.01 ± 0.02	−0.29 ± 0.21	−0.64	0.04

^a^ σ_P_^2^ = phenotypic variance; h_d_^2^ = direct heritability; h_m_^2^ = maternal heritability; c^2^ = proportion of phenotypic variance due to the permanent environmental effect of the dam; r_am_ = correlation between direct and maternal genetic effects; cov_am_ = covariance between direct and maternal genetic effects. ^b^ IGF28, IGF42, and IGF56 are the IGF-I concentrations on days 28, 42, and 56 of the 140-day postweaning test. ^c^ Mean IGF-I is the average of serum IGF-I measurements taken on days 28, 42, and 56 of the postweaning period for each calf.

**Table 4:** Genetic, environmental, and phenotypic correlations ^a^ among IGF-I measurements at weaning (IGFWEAN), day 28 (IGF28), 42 (IGF42), and 56 (IGF56) of the postweaning performance test, and mean IGF-I concentration derived from reduced bivariate models.

IGF-I Measurement					
IGF-I Measurement	Correlation	IGF28	IGF42	IGF56	Mean IGF-I ^b^
IGF Wean	r_A1A2_	0.90 ^c^	0.82	0.63	0.69
	r_A1M2_	−0.64	−0.43	−0.36	−0.32
	r_A2M2_	−0.91	−0.87	−0.89	−0.91
	r_E1E2_	0.38	0.27	0.26	0.33
	r_P1P2_	0.52	0.45	0.37	0.45
IGF28	r_A1A2_		0.94 ± 0.05	0.93 ± 0.04	0.98 ± 0.01
	r_A1M1_		−0.23 ± 0.20	−0.91 ± 0.05	−0.89 ± 0.05
	r_A1M2_		0.01 ± 0.21	−0.82 ± 0.12	−0.86 ± 0.08
	r_A2M1_		−0.51 ± 0.23	−0.88 ± 0.10	−0.87 ± 0.07
	r_A2M2_		−0.31 ± 0.20	−0.89 ± 0.07	−0.86 ± 0.06
	r_M1M2_		0.97 ± 0.09	0.95 ± 0.09	0.99 ± 0.03
	r_E1E2_		0.59 ± 0.02	0.45 ± 0.03	0.80 ± 0.02
	r_P1P2_		0.69	0.59	0.86
IGF42	r_A1A2_			0.89 ± 0.05	0.97 ± 0.01
	r_A1M1_			−0.91 ± 0.05	−0.91 ± 0.04
	r_A1M2_			−0.78 ± 0.11	−0.91 ± 0.06
	r_A2M1_			−0.69 ± 0.11	−0.79 ± 0.07
	r_A2M2_			−0.87 ± 0.06	−0.87 ± 0.05
	r_M1M2_			0.74 ± 0.09	0.93 ± 0.03
	r_E1E2_			0.56 ± 0.03	0.67 ± 0.01
	r_P1P2_			0.65	0.89
IGF56	r_A1A2_				0.96 ± 0.02
	r_A1M1_				−0.87 ± 0.07
	r_A1M2_				−0.85 ± 0.08
	r_A2M1_				−0.83 ± 0.09
	r_A2M2_				−0.90 ± 0.05
	r_M1M2_				0.93 ± 0.04
	r_E1E2_				0.82 ± 0.01
	r_P1P2_				0.85

^a^ r_A1A2_ = additive genetic correlation between traits 1 and 2; r_A1M1_ = correlation between additive and maternal genetic effects of trait 1; r_A1M2_ = correlation between additive genetic effect of trait 1 and maternal genetic effect of trait 2; r_A2M1_ = = correlation between additive genetic effect of trait 2 and maternal genetic effect of trait 1; r_A2M2_ = correlation between additive and maternal genetic effects of trait 2; r_M1M2_ = correlation between the maternal genetic effects of trait 1 and 2; r_E1E2_ = environmental correlation between trait 1 and 2, and r_P1P2_ = phenotypic correlation between trait 1 and 2. ^b^ Mean IGF-I = average of serum IGF-I measurements taken at days 28, 42, and 56 of the postweaning period for each calf. ^c^ Standard errors of the correlations between IGF-I at weaning and postweaning IGF-I measures could not be calculated by the MTDFREML software [12] because blood samples were collected at weaning and during the postweaning period only for calves born in 2003.

**Table 5:** Genetic, environmental, and phenotypic correlations ^a^ of IGF-I measurements at weaning (IGFWEAN), day 28 (IGF28), 42 (IGF42), and 56 (IGF56) of the postweaning performance test and mean IGF-I concentration with weight traits derived from reduced bivariate models.

Weight Trait								
IGF-I Measurement	Correlation	Birth wt	Weaning wt	On-Test wt	d-28 wt	d-42 wt	d-56 wt	Off-Test wt
IGFWEAN	r_A1A2_	−0.23 ± 0.28	−0.12 ± 0.33	−0.12 ^c^	−0.09	0.07	−0.03	0.14
	r_A1M2_	0.57 ± 0.35	0.63 ± 0.26	0.74	0.76	0.72	0.77	0.95
	r_A2M2_	−0.36 ± 0.44	−0.38 ± 0.29	−0.42	−0.29	−0.26	−0.30	−0.18
	r_E1E2_	−0.08 ± 0.10	0.56 ± 0.13	0.71	0.76	0.66	0.72	0.35
	r_P1P2_	−0.06	0.35	0.46	0.48	0.49	0.49	0.35
IGF28	r_A1A2_	−0.26 ± 0.14	0.12 ± 0.13	0.05 ± 0.13	0.13 ± 0.13	0.13 ± 0.13	0.12 ± 0.13	0.20 ± 0.12
	r_A1M1_	−0.91 ± 0.05	−0.90 ± 0.04	−0.90 ± 0.05	−0.89 ± 0.05	−0.90 ± 0.05	−0.89 ± 0.05	−0.91 ± 0.04
	r_A1M2_	0.37 ± 0.16	−0.04 ± 0.20	0.11 ± 0.22	0.06 ± 0.23	0.12 ± 0.24	0.15 ± 0.23	0.23 ± 0.37
	r_A2M1_	0.10 ± 0.17	−0.13 ± 0.15	0.02 ± 0.17	−0.06 ± 0.16	−0.09 ± 0.15	−0.07 ± 0.17	−0.20 ± 0.15
	r_A2M2_	−0.30 ± 0.15	−0.58 ± 0.12	−0.56 ± 0.15	−0.48 ± 0.17	−0.41 ± 0.17	−0.45 ± 0.17	−0.32 ± 0.28
	r_M1M2_	−0.26 ± 0.18	0.26 ± 0.21	0.06 ± 0.23	0.15 ± 0.24	0.12 ± 0.23	0.09 ± 0.24	0.09 ± 0.36
	r_E1E2_	−0.01 ± 0.05	0.04 ± 0.07	0.16 ± 0.05	0.17 ± 0.05	0.21 ± 0.06	0.19 ± 0.05	0.15 ± 0.05
	r_P1P2_	−0.08	0.09	0.13	0.16	0.18	0.17	0.17
IGF42	r_A1A2_	−0.08 ± 0.14	0.11 ± 0.12	0.04 ± 0.13	0.12 ± 0.13	0.08 ± 0.13	0.12 ± 0.13	0.15 ± 0.03
	r_A1M1_	−0.91 ± 0.04	−0.90 ± 0.04	−0.90 ± 0.05	−0.90 ± 0.05	−0.90 ± 0.05	−0.90 ± 0.05	−0.90 ± 0.05
	r_A1M2_	0.31 ± 0.16	−0.11 ± 0.22	0.09 ± 0.23	0.00 ± 0.25	0.13 ± 0.25	0.04 ± 0.25	−0.06 ± 0.44
	r_A2M1_	−0.03 ± 0.16	−0.11 ± 0.15	0.02 ± 0.16	−0.02 ± 0.16	−0.03 ± 0.15	−0.07 ± 0.16	−0.08 ± 0.16
	r_A2M2_	−0.31 ± 0.15	−0.60 ± 0.13	−0.60 ± 0.16	−0.52 ± 0.19	−0.45 ± 0.18	−0.49 ± 0.18	−0.41 ± 0.32
	r_M1M2_	−0.26 ± 0.16	0.27 ± 0.22	0.02 ± 0.24	0.14 ± 0.25	0.12 ± 0.26	0.18 ± 0.25	0.28 ± 0.41
	r_E1E2_	−0.06 ± 0.06	0.04 ± 0.07	0.14 ± 0.06	0.16 ± 0.06	0.22 ± 0.06	0.20 ± 0.05	0.17 ± 0.05
	r_P1P2_	−0.07	0.08	0.11	0.15	0.18	0.17	0.16
IGF56	r_A1A2_	−0.29 ± 0.14	0.19 ± 0.14	0.19 ± 0.14	0.26 ± 0.14	0.18 ± 0.14	0.21 ± 0.14	0.26 ± 0.14
	r_A1M1_	−0.88 ± 0.07	−0.88 ± 0.09	−0.88 ± 0.07	−0.88 ± 0.07	−0.88 ± 0.06	−0.88 ± 0.07	−0.89 ± 0.05
	r_A1M2_	0.48 ± 0.16	−0.09 ± 0.23	−0.05 ± 0.25	−0.04 ± 0.26	0.18 ± 0.27	0.12 ± 0.26	0.11 ± 0.43
	r_A2M1_	0.15 ± 0.21	−0.15 ± 0.21	−0.14 ± 0.20	−0.18 ± 0.20	−0.19 ± 0.17	−0.20 ± 0.20	−0.30 ± 0.17
	r_A2M2_	−0.31 ± 0.15	−0.60 ± 0.13	−0.59 ± 0.16	−0.50 ± 0.18	−0.42 ± 0.18	−0.47 ± 0.18	−0.36 ± 0.30
	r_M1M2_	−0.38 ± 0.20	0.12 ± 0.29	0.13 ± 0.29	0.13 ± 0.31	−0.01 ± 0.29	0.05 ± 0.30	0.05 ± 0.43
	r_E1E2_	0.00 ± 0.05	−0.01 ± 0.06	0.04 ± 0.05	0.05 ± 0.05	0.12 ± 0.05	0.11 ± 0.05	0.07 ± 0.05
	r_P1P2_	−0.04	0.06	0.08	0.11	0.13	0.14	0.12
Mean IGF-I ^b^	r_A1A2_	−0.17 ± 0.13	0.12 ± 0.12	0.05 ± 0.13	0.14 ± 0.12	0.13 ± 0.12	0.13 ± 0.12	0.18 ± 0.12
	r_A1M1_	−0.90 ± 0.04	−0.90 ± 0.04	−0.90 ± 0.05	−0.90 ± 0.05	−0.90 ± 0.04	−0.90 ± 0.05	−0.91 ± 0.04
	r_A1M2_	0.32 ± 0.15	−0.02 ± 0.20	0.14 ± 0.22	0.10 ± 0.23	0.18 ± 0.24	0.17 ± 0.23	0.27 ± 0.41
	r_A2M1_	0.04 ± 0.17	−0.10 ± 0.15	0.04 ± 0.16	−0.03 ± 0.16	−0.08 ± 0.15	−0.05 ± 0.16	−0.16 ± 0.15
	r_A2M2_	−0.31 ± 0.15	−0.59 ± 0.13	−0.58 ± 0.16	−0.49 ± 0.18	−0.42 ± 0.18	−0.46 ± 0.17	−0.33 ± 0.31
	r_M1M2_	−0.25 ± 0.17	0.15 ± 0.21	−0.04 ± 0.23	0.03 ± 0.25	0.03 ± 0.24	0.01 ± 0.24	−0.02 ± 0.39
	r_E1E2_	−0.04 ± 0.05	0.04 ± 0.07	0.14 ± 0.06	0.15 ± 0.06	0.22 ± 0.06	0.19 ± 0.05	0.15 ± 0.06
	r_P1P2_	−0.08	0.08	0.11	0.15	0.19	0.17	0.16

^a^ r_A1A2_ = additive genetic correlation between traits 1 and 2; r_A1M1_ = correlation between additive and maternal genetic effects of trait 1; r_A1M2_ = correlation between additive genetic effect of trait 1 and maternal genetic effect of trait 2; r_A2M1_ = = correlation between additive genetic effect of trait 2 and maternal genetic effect of trait 1; r_A2M2_ = correlation between additive and maternal genetic effects of trait 2; r_M1M2_ = correlation between the maternal genetic effects of trait 1 and 2; r_E1E2_ = environmental correlation between trait 1 and 2, and r_P1P2_ = phenotypic correlation between trait 1 and 2. ^b^ Mean IGF-I = average of serum IGF-I measurements taken at days 28, 42, and 56 of the postweaning period for each calf. ^c^ Standard errors of the correlations between IGF-I at weaning and the postweaning traits could not be calculated by the MTDFREML software because these traits were only available for calves born in 2003.

**Table 6:** Genetic, environmental, and phenotypic correlations ^a^ of IGF-I measurements at weaning (IGFWEAN), day 28 (IGF28), 42 (IGF42), and 56 (IGF56) of the postweaning performance test, and mean IGF-I with postweaning weight gain and off-test hip height derived from reduced bivariate models.

Trait			
IGF-I Measurement	Correlation	Postweaning Gain	Off-Test Hip Height
IGFWEAN	r_A1A2_	0.45 ^c^	0.24
	r_A1M2_	0.07	0.87
	r_A2M2_	−0.86	−0.27
	r_E1E2_	0.12	0.19
	r_P1P2_	0.12	0.28
IGF28	r_A1A2_	0.18 ± 0.13	0.27 ± 0.11
	r_A1M1_	−0.92 ± 0.04	−0.90 ± 0.04
	r_A1M2_	0.15 ± 0.35	0.13 ± 0.22
	r_A2M1_	−0.22 ± 0.15	−0.14 ± 0.13
	r_A2M2_	−0.94 ± 0.21	−0.20 ± 0.16
	r_M1M2_	−0.04 ± 0.35	0.06 ± 0.23
	r_E1E2_	0.10 ± 0.05	0.04 ± 0.06
	r_P1P2_	0.12	0.14
IGF42	r_A1A2_	0.12 ± 0.13	0.22 ± 0.11
	r_A1M1_	−0.90 ± 0.05	−0.89 ± 0.05
	r_A1M2_	−0.27 ± 0.37	−0.02 ± 0.23
	r_A2M1_	0.02 ± 0.16	−0.04 ± 0.14
	r_A2M2_	−0.96 ± 0.30	−0.22 ± 0.17
	r_M1M2_	0.16 ± 0.40	0.11 ± 0.24
	r_E1E2_	0.15 ± 0.05	0.06 ± 0.06
	r_P1P2_	0.15	0.13
IGF56	r_A1A2_	0.18 ± 0.14	0.32 ± 0.12
	r_A1M1_	−0.89 ± 0.06	−0.87 ± 0.06
	r_A1M2_	0.21 ± 0.36	0.23 ± 0.23
	r_A2M1_	−0.21 ± 0.18	−0.17 ± 0.16
	r_A2M2_	−0.88 ± 0.19	−0.22 ± 0.15
	r_M1M2_	−0.28 ± 0.41	−0.27 ± 0.25
	r_E1E2_	0.12 ± 0.05	0.00 ± 0.06
	r_P1P2_	0.12	0.11
Mean IGF-I ^b^	r_A1A2_	0.13 ± 0.12	0.25 ± 0.11
	r_A1M1_	−0.91 ± 0.04	−0.90 ± 0.04
	r_A1M2_	0.19 ± 0.35	0.15 ± 0.21
	r_A2M1_	−0.11 ± 0.15	−0.08 ± 0.13
	r_A2M2_	−0.94 ± 0.22	−0.20 ± 0.15
	r_M1M2_	−0.17 ± 0.36	−0.06 ± 0.22
	r_E1E2_	0.15 ± 0.05	0.04 ± 0.06
	r_P1P2_	0.14	0.14

^a^ r_A1A2_ = additive genetic correlation between traits 1 and 2; r_A1M1_ = correlation between additive and maternal genetic effects of trait 1; r_A1M2_ = correlation between additive genetic effect of trait 1 and maternal genetic effect of trait 2; r_A2M1_ = = correlation between additive genetic effect of trait 2 and maternal genetic effect of trait 1; r_A2M2_ = correlation between additive and maternal genetic effects of trait 2; r_M1M2_ = correlation between the maternal genetic effects of trait 1 and 2; r_E1E2_ = environmental correlation between trait 1 and 2, and r_P1P2_ = phenotypic correlation between trait 1 and 2. ^b^ Mean IGF-I = average of serum IGF-I measurements taken on days 28, 42, and 56 of the postweaning period for each calf. ^c^ Standard errors of the correlations between IGF-I at weaning and postweaning traits could not be calculated by the MTDFREML software because these traits were only available for calves born in 2003.

## Data Availability

Contact the corresponding author for inquiries concerning data availability.
